# Relapsing toxic epidermal necrolysis following COVID-19

**DOI:** 10.1016/j.jdcr.2024.07.011

**Published:** 2024-07-31

**Authors:** Feben Messele, Luke Horton, Ajay N. Sharma, Michelle S. Min, Nathan W. Rojek, Kenneth G. Linden

**Affiliations:** Department of Dermatology, University of California, Irvine, Irvine, California

**Keywords:** COVID-19, relapsing, SJS, Stevens-Johnson syndrome, TEN, toxic epidermal necrolysis

## Background

Stevens-Johnson syndrome (SJS) and toxic epidermal necrolysis (TEN) are dermatologic emergencies classically triggered by medications, most commonly: antibiotics, antiepileptics, nonsteroidal antiinflammatory drugs, allopurinol, and immune checkpoint inhibitors.^1^ Infection may also be an inciting factor, with COVID-19 particularly associated with an increase in inflammatory cytokines, thereby heightening patient susceptibility to SJS/TEN.^2^ This is highlighted by the reported 2-fold increase in incidence of SJS/TEN after COVID-19.^3^ Clinical features of SJS/TEN include widespread, diffuse erythema and/or dusky papules coalescing into plaques that usually progress into flaccid bullae with skin sloughing. Mucosal tissue is usually involved, and a positive Nikolsky sign may be present.^1^

Currently, the pathophysiology of SJS/TEN is not well understood, with a T-cell mediated type IV hypersensitivity to drug metabolites proposed as the most likely mechanism.^1^ Here, we present a challenging case of refractory and relapsing TEN without a clear causative medication, thought to be secondary to immune sequela following COVID-19.

## Case report

**Fig 1:**
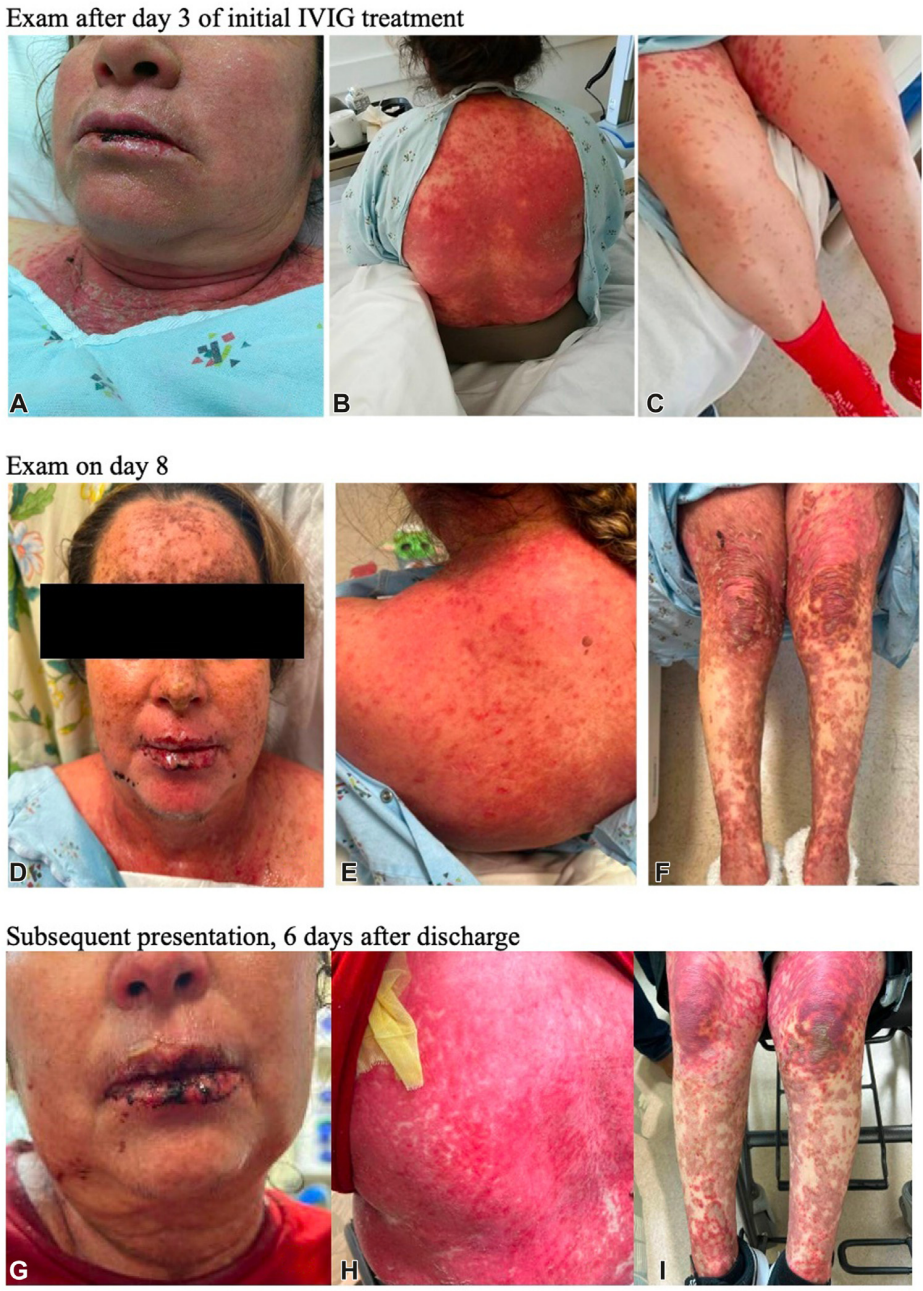
Examination after day 3 of initial IVIG treatment. Diffuse morbilliform eruption with bullae formation overlying dusky skin necrosis involved the (**A**) mucosal lips, neck, chest, (**B**) back, and (**C**) extremities. Examination on day 8. Dusky and necrosed papules coalescing into plaques with overlying flaccid and hemorrhagic bullae are seen on (**D**) the face and mucosal lips, (**E**) trunk including her back, and (**F**) extremities. Subsequent presentation, 6 days after discharge from initial presentation. **G,** Hemorrhagic crust on the lips. **H,** Diffuse erythroderma and erosions on the back with skin sloughing. **I,** Dusky papules coalescing into plaques with overlying flaccid and hemorrhagic bullae on the lower extremities.

A 48-year-old woman with a history of unicentric Castleman disease (treated 6 months before presentation with rituximab, siltuximab, and radiation) presented with a worsening pruritic and erythematous rash on the face, extremities, back, and trunk comprising about 20% of her body surface area. Four weeks prior, she had contracted COVID-19 as confirmed by an at home antigen test for which she began a 5-day course of nirmatrelvir/ritonavir (Paxlovid), taken 3 weeks before presentation. Besides Paxlovid, she denied taking any medications or supplements. Examination showed a positive Nikolsky sign. Two biopsies revealed necrotic keratinocytes consistent with early-stage SJS/TEN. Her calculated severity-of-illness score for toxic epidermal necrolysis score was 5 at this time (mortality rate > 90%). The patient was given 2 g/kg of intravenous immunoglobulin (IVIG) over 3 days, and 3 mg/kg/d of cyclosporine. Examination after day 3 of IVIG showed progression of her rash with worsening erosions on the lower lip, edema, and scale on the ears, and dusky morbilliform papules coalescing into plaques on her torso (Fig 1). The patient was afebrile with mild transaminitis (ALT 59 U/L) and eosinophilia (600 cells/mcL). Viral workup (human herpes virus6/7, Ebstein-Barr virus, cytomegalovirus, and herpes simplex virus) and mycoplasma immunoglobulin-M were negative.

**Fig 2:**
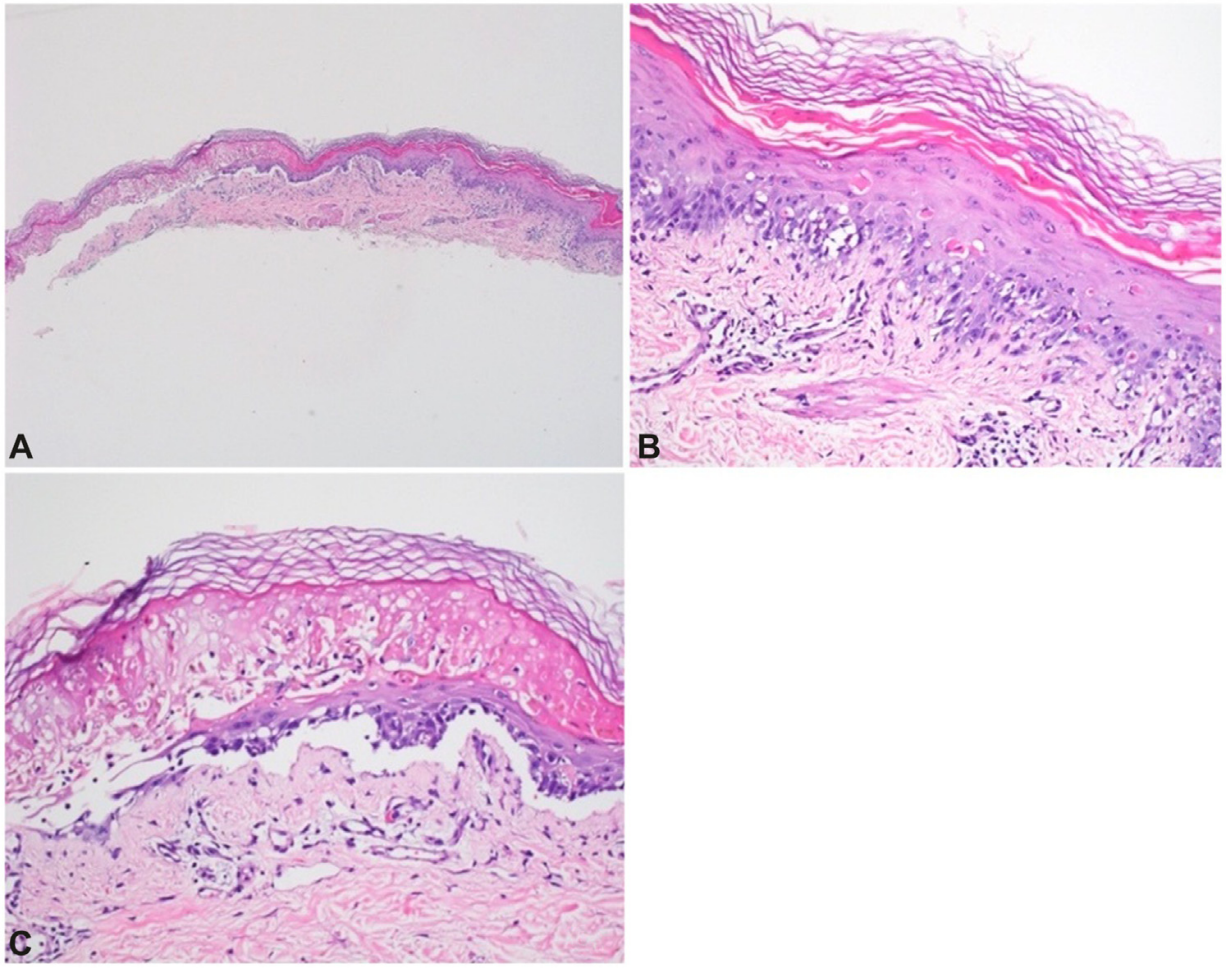
Shave biopsy revealed (**A, C**) a subepidermal split with areas of full-thickness epidermal necrosis and (**B, C**) interface dermatitis with scattered necrotic keratinocytes. (**A-C,** Hematoxylin-eosin stain; original magnifications: **A,** ×4; **B** and **C,** ×40.)

The patient then started 60 mg daily of prednisone and topical corticosteroids. By day 8, the patient continued to worsen with bullae formation, sloughing skin, and oral erosions (Fig 1). Shave biopsy showed an intraepidermal split with areas of full-thickness necrosis and areas of scattered necrotic keratinocytes consistent with TEN (Fig 2). Given her worsening clinical status, the patient was started on a second round of IVIG 3 g/kg delivered over 4 days and 3 mg/kg/d of cyclosporine with a rapid taper of her prednisone. On this regimen, the patient showed significant improvement and was discharged on day 21.

Six days after discharge, the patient presented to the Emergency Department with worsening skin pain and full-body rash with skin sloughing (Fig 1). The patient denied taking any medications including nonsteroidal antiinflammatory drugs. Expanded workup with a pemphigoid antibody panel, pemphigus antibody panel, anticollagen VII antibody, and direct immunofluorescence were negative. Repeat biopsy showed an interface dermatitis with epidermal necrosis consistent with TEN.

The patient was started on her third cycle of IVIG 3 g/kg/d within the month and 5.0 mg/kg/d of cyclosporine, which failed to improve her symptoms. Because of her initial improvement with infliximab, a second dose was administered 1 week after the first. When she failed to respond to treatment, a fourth cycle of IVIG 3 g/kg was administered.

The patient continued to decompensate, requiring antibiotics, vasopressors, and eventually intubation because of acute hypoxemic respiratory failure. Tragically, despite maximal supportive efforts, the patient passed away a month into her second hospitalization.

## Discussion

COVID-19 has been associated with SJS/TEN, with a 7-fold increase in cases during the COVID-19 pandemic.^4^ The pathophysiology is not fully understood, although Stanley et al^4^ proposed 3 possible mechanisms for SJS/TEN in the setting of COVID-19: vaccine-induced, virus-induced, and a threshold-lowering pathway, increasing drug hypersensitivity. Given our patient’s COVID-19 vaccination was over 2 years ago, vaccination was an unlikely culprit. Her recent COVID-19, however, with poor clearance of viral inflammatory milieu, could explain her immune dysregulation leading to TEN. Furthermore, viruses bind to major histocompatability complex I on antigen-presenting cells, activating cytotoxic T cells, inducing SJS/TEN.^4^ Increased drug hypersensitivity secondary to COVID-19, coupled with direct effects of Paxlovid is another possible mechanism. Malviya et al^5^ attributed a case of SJS in a patient on Paxlovid (nirmatrelvir/ritonavir) for COVID-19 to ritonavir, whereas Lowndes et al^6^ recounted SJS in a patient within days of treatment with rituximab. Alden score, drug causality assessment for SJS/TEN yielded unlikely association (score ≤ 1) for both Paxlovid (1) and rituximab (–1). The most common treatments demonstrating survival benefits in TEN patients include cyclosporine, IVIG, and tumor necrosis factor-alfa inhibitors, however, as this case illustrates, response is variable and more reliable treatment options are necessary.^7^

Other diagnoses can be considered including acute cutaneous lupus erythematosus, acute pan-epidermolysis, reactive infectious mucosal eruption, and paraneoplastic pemphigus (PNP). With an antinuclear antibody titer never greater than 1:40, this patient did not meet the latest European League against Rheumatism/American College of Rheumatology criteria for SLE. Direct immunofluorescence also did not support lupus. Acute pan-epidermolysis and reactive infectious mucosal eruption may be considered though SJS/TEN was favored given the clinical presentation and course.^8^ Castleman’s potentially causing PNP was considered, however direct immunofluorescence was performed with negative results. PNP is associated with 10% of Castleman disease cases.^9^ The 3 major criteria to diagnose PNP include polymorphic mucocutaneous eruptions, concomitant neoplasm, and serum antibodies with a specific immunoprecipitation pattern.^10^ Patient meets 2 of these criteria, however with both direct and indirect immunofluorescence yielding negative test results, SJS/TEN was favored.

Multiple therapies, including systemic corticosteroids, IVIG, cyclosporine, and infliximab, were used throughout our patient’s disease course without sustained improvement. Despite the cessation of all medications, our patient reflared after significant improvement without a clear drug culprit, eventually leading to her unfortunate passing. We posit that the patients’ COVID-19 may have induced a relapsing TEN.

Our case highlights the severe immunocutaneous sequelae that may result from COVID-19, emphasizing the need for further research to understand the interplay between the pathophysiology of COVID-19 with hopes of developing more targeted therapies.

## Conflicts of interest

Dr Min is on the advisory boards of Horizon, McGraw Hill, and BMS and is an investigator for Amgen, BI, MBS, and Priovant. Dr Rojek has served on the advisory board of Boehringer Ingelheim. The other authors have no conflicts of interest to declare.
